# Smell perception in normal tension glaucoma patients

**Published:** 2010-03-23

**Authors:** Maneli Mozaffarieh, Daniela Hauenstein, Andreas Schoetzau, Katarzyna Konieczka, Josef Flammer

**Affiliations:** Department of Ophthalmology, University Hospital Basel, Basel, Switzerland

## Abstract

**Purpose:**

The aim of this study was to quantify the ability to identify odors in normal tension glaucoma (NTG) patients and healthy subjects with and without a primary vascular dysregulation (PVD).

**Methods:**

Both self-assessment of smell perception and evaluation of odor identification by means of the 12-item odor identification test (“Sniffin’ Sticks”) were performed in the following groups of subjects: 1) 18 NTG patients with PVD (G+), 2) 18 NTG patients without PVD (G-), 3) 18 healthy subjects with PVD (H^+^) and 4) 18 healthy subjects without PVD (H-). The subjects self-assessment of smell perception was evaluated before the Sniffin’ Sticks test by asking them to judge their ability to identify odors as either “average,” “better than average,” or “worse than average.”

**Results:**

Subjects with a PVD (G+ and H^+^) can identify odors significantly better than those without a PVD (G- and H-; in a score scale of 1–12 the score point difference=2.64, 95% CI=1.88–3.40, p<0.001). No significant differences in odor identification was found between NTG (groups G+ and G-) and healthy subjects (groups H^+^ and H-; score point difference=-0.14, 95% CI=-0.9–0.62, p=0.72).

**Conclusions:**

Subjects with a PVD can identify odors significantly better than those without a PVD.

## Introduction

Smell perception can have a significant impact on our lives [1]. When the sense of smell is lost, it is not just that we cannot differentiate between different smells or enjoy what we eat or drink but we are also not as alert to dangers [2]. Similarly, an enhanced perception of the sense of smell may be just as disturbing [3,4]. The increased sense of smell to different odors can be overwhelming enough to cause nausea, sneezing, headaches or eye pain [5,6].

Disturbances in smell perception are not infrequent [7,8], particularly in patients with neurodegenerative diseases such as Parkinson disease [9], for instance. Olfactory dysfunction among subjects below 65 years of age is more frequent than previously reported [10]. Patients often complain to their physicians about both disturbances of hypo- and hyperosmia [11]. We have had similar experiences with normal tension glaucoma patients (NTG). Our clinical observations implied that those patients with the better sense of smell had a primary vascular dysregulation (PVD).

PVD is an inborn predisposition to respond different to various stimuli [12,13]. PVD individuals tend to more often have: cold hands or feet even in the summer [14], a reduced feeling of thirst [15], a low blood pressure especially when they are young [16], a longer sleep onset time [17], migraines in comparison to non-PVD subjects [18], and an altered drug sensitivity due to differential expression of ABC transporter proteins [19].

Other leading signs of PVD include increased level of Endothelin-1 [20], blood-pressure dependent Endothelin-sensitivity [21], and an increased prevalence of silent myocardial ischemia [22]. PVD subjects also have a higher risk for certain diseases. This includes the risk of anterior ischemic optic neuropathy (AION) [23], vein occlusions [24], central serous chorioretinopathy [25,26], as well as glaucoma [27,28].

In terms of circulation, PVD subjects respond more strongly with vasoconstriction to mechanical stress (e.g., whip lash trauma), psychological stress, or coldness [12]. Analysis of their retinal circulation has shown that these subjects respond less to flickering light (neurovascular coupling) [29], they have increased spatial irregularities [30], fast pulse waves indicating higher stiffness of vessels [31], and altered blood-brain and blood-retinal barrier including increased prevalence of splinter hemorrhages [32]. In PVD subjects relationships between finger circulation and visual field [14], finger circulation and ophthalmic artery blood flow [33], as well as between corneal temperature and finger temperature have been described [34]. The aim of the present study was to examine whether NTG or PVD subjects have a different ability to smell. We therefore quantified the ability to identify odors in four groups of subjects, namely, NTG patients with and without PVD as well as healthy subjects with and without PVD.

## Methods

### Participants

Patients with normal tension glaucoma (NTG) were recruited from the University Eye Clinic Basel (Basel, Switzerland) between January 2009 and December 2009. Healthy volunteers, age and sex matched to NTG patients, were recruited after a notification in the University Clinic informed potential volunteers of the opportunity to participate in a scientific research project. Ethical approval was obtained from the local medical ethics committee, and written informed consent was received from all subjects before entry into the study. The study was designed and conducted in accordance with the tenets of Declaration of Helsinki.

Patients with NTG had to meet the following inclusion criteria: (1) untreated intraocular pressure (IOP) less than 21 mmHg on multiple measurements, (2) progressive changes in either visual fields or optic nerve cupping, and (3) the absence of other known causes of optic neuropathy than glau coma.

PVD was defined as being present (PVD+) if the subjects answered three of the following seven questions with “Yes,” and it was defined as being absent (PVD-) if the subjects answered less than three questions with “Yes”: 1) Do you suffer from cold hands or feet even in summer [14], 2) Do you have trouble falling asleep, especially when you are cold [17], 3) Are you seldom thirsty and do you have to remind yourself to drink enough [15], 4) Do you suffer from tinnitus [13], 5) Do you suffer from migraine attacks [18], 6) Do you have a rather low blood pressure [16], 7) Do you react sensitively to certain medications [19]. Demographic data of the different groups of participants are given in Table 1.

**Table 1:** Demographic data of the different groups of participants.

** **	** **	** **	**Gender**			
** **	** **	** **	**Male**		**Female**	
**Group**	**Mean age**	**N**	**N**	**%**	**N**	**%**
G+	61.6	18	9	50	9	50
G-	58.5	18	3	17	15	83
H^+^	56.3	18	10	56	8	44
H-	53.4	18	9	50	9	50

Demographic data of the different groups of participants showing number (N) of participants in each group, mean age and gender distribution. G+=NTG patients with PVD; G-=NTG patients without PVD; H^+^=Healthy subjects with PVD; H-=Healthy subjects without PVD; PVD=Primary vascular dysregulation.

### Self-assessment

All participants rated their perception of smell as either “average,” “better than average” or “worse than average” before the Sniffin’ Sticks test (Burghart GmbH,  Wedel, Germany) was performed.

### Sniffin’ Sticks test

The 12-item “Sniffin’ Sticks” test battery is a validated test for the evaluation of identification of odors [35]. This olfactory test kit consisted of pen-like odor-dispensing devices (“Sniffin’ Sticks”). Twelve odors were presented in a randomized sequence. Subjects were allowed to sample the odors one time before identifying them from a list of four descriptors. The test score which varied from 0 (worst) to 12 (best) was the sum of the correctly identified odors. The experimenter (DH) was not informed as to which group the test person belonged to. She (DH) presented odor pens to both nostrils of participants separated by an interval of 20–30 s to prevent olfactory desensitization.

### Statistical analysis

To predict score points from NTG status and PVD status, a linear regression model was performed. Independent variables were NTG status and PVD status as well as an interaction between both variables. Results are presented as differences of means between PVD+ and PVD- groups as well as differences between NTG and healthy groups. Differences were estimated with corresponding 95% confidence intervals (CI). To predict score points from self-assessment a linear model was also used. A p value <0.05 is considered as significant. All analyses were done using the statistical package R version 2.4.0 (SPSS 2006, Basel, Switzerland).

## Results

**Figure 1:**
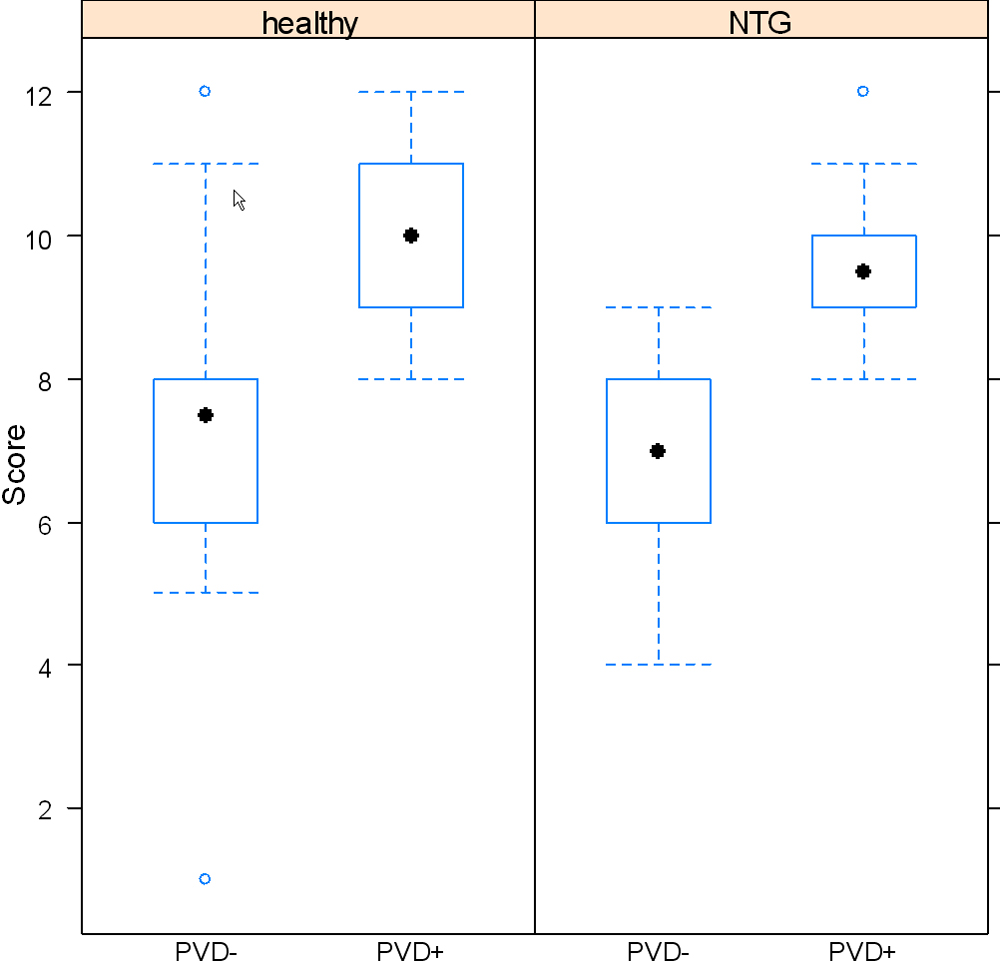
Sniffin’ Sticks score results in healthy and normal tension glaucoma (NTG) subjects. Healthy subjects with a PVD as well as glaucoma patients with a PVD could identify odors better than those without a PVD.

Results of the subject’s self-assessment of smell perception before Sniffin’ Sticks test are given in Table 2. There was no significant interaction between NTG and PVD (p=0.94), indicating the same score difference in each PVD group. Consequently, the interaction was deleted from the regression model. Subjects with a PVD (G+ and H^+^) could identify odors significantly better than those without a PVD (G- and H-; p<0.001; Figure 1). No significant differences in odor identification was found between NTG (G+ and G-) and healthy subjects (H^+^ and H-; p=0.72). No change was observed after correcting for age. Subjects with a better self assessment before Sniffin’ Sticks test had significantly higher score points (p<0.001; Table 3).

**Table 2:** Self-assessment of smell perception before Sniffin’ Sticks test. Scores are given in %.

**Group**	**N**	**Average %**	**Better than average %**	**Worse than average %**
G+	18	39	50	11
G-	18	56	0	44
H^+^	18	33	61	6
H-	18	11	11	78

This table depicts the subject’s self-assessment of their smell perception prior to the Sniffin’ Sticks test. The participants were asked to judge their ability to smell as either “average”,  “better than average” or “worse than average". In the table, G+=NTG patients with PVD; G-=NTG patients without PVD; H^+^=Healthy subjects with PVD; H-=Healthy subjects without PVD; PVD=Primary vascular dysregulation.

**Table 3:** Sniffin’ Sticks Score results in the four different groups of participants.

** **	**Sniffin’ Sticks Score (range 0–12)**			
**Group**	**Mean**	**Median**	**Minimum**	**Maximum**
G+	9.7	9.5	8	12
G-	7.0	7.0	4.0	9.0
H^+^	9.8	10.0	8.0	12.0
H-	7.2	7.5	1.0	12.0

G+=NTG patients with PVD; G-=NTG patients without PVD; H^+^=Healthy subjects with PVD; H-=Healthy subjects without PVD; PVD=Primary vascular dysregulation.

## Discussion

In the present study we found that both NTG patients and healthy individuals having a PVD are able to identify various odors better than those without a PVD. As previously mentioned, individuals with a PVD on average tend to respond different to different stimuli [13]. When drugs, for example, are prescribed to PVD subjects they often respond stronger, at times even violently to certain class of drugs. Similarly, they respond less than the average person to a few other classes of drugs. This might be due to a different expression of the ABC transport proteins in these PVD individuals [19].

Proteins also play a role in smell perception. Odorant binding proteins, which are found in the human olfactory mucus [36], bind to different odorants. They are small abundant extracellular proteins belonging to the lipocalin superfamily [37-39]. These proteins, secreted by the olfactory epithelium in the nasal mucus of vertebrates, are carrier proteins [40]. It is thought that these proteins act as lipophilic ligands which transfer odorants across the mucous layer to the receptors and thereby increase the concentration of the odorants in the layer, relative to air [41-43]. A differential expression of these odorant binding proteins, similar to the differential expression found in ABC transport proteins, may be one explanation for the altered smell perception in PVD subjects.

Olfactory genes form the largest multi-gene family in humans [44,45]. These genes encode olfactory receptors, which interact with odorant molecules in the nose to initiate a neuronal response that triggers the perception of smell. These olfactory receptors are G protein-coupled receptors; upon odorant binding, these receptors couple to G proteins, resulting in an increase in intracellular cAMP levels and subsequent receptor signaling [46]. The altered smell perception in PVD subjects could potentially also be due to an altered expression of these receptors.

As with the TAS2R50 bitter receptor gene, a single nucleotide polymorphism in the *OR13G1* (olfactory receptor, family 13, subfamily G, member 1) gene has been linked with an increased risk of myocardial infarction [47]. This olfactory receptor gene may play an indirect role in increasing the risk of myocard infarction by affecting food preferences that are determined by the sense of smell. Similarly, we could argue an olfactory receptor gene may play an indirect role in increasing the risk for a PVD syndrome.

In conclusion, subjects with a PVD can identify odors significantly better than those without a PVD. We do not know the cause of this different smell perception and can only speculate on it. Further research on the role of odorant-binding proteins and the genetics of olfactory receptors is warranted.
